# Supplementary material for: Unveiling the Role of RNA Recognition Motif Proteins in Orchestrating Nucleotide-Binding Site and Leucine-Rich Repeat Protein Gene Pairs and Chloroplast Immunity Pathways: Insights into Plant Defense Mechanisms
Source: Int J Mol Sci. 2024 May 20;25(10):5557. doi: 10.3390/ijms25105557 (PMC11122538; doi:10.3390/ijms25105557)
Supplement: Supplementary file 1 [file ijms-25-05557-s001.zip › ijms-2996889-supplementary.pdf]

Supplementary Table S1. Primers used in this study.

Name	Sequence	Purpose
ko-*OsRRM2* F	gccGAGAAGAGCGTGGCCGCCAT	Adaptor for Cas9 system
ko-*OsRRM2* R	aaacATGGCGGCCACGCTCTTCTC	Adaptor for Cas9 system
gfp-*OsRRM2* F	ggagaggacagcccagatcaactagtATGGCGGCCACGC	HR-PCR for pAN580
gfp-*OsRRM2* R	tcctcgcccttgctcaccatggatccATATTGTCTCCTAGGTGGTTTCG	HR-PCR for pAN580
gfp-*Pik1-H4* F	ggagaggacagcccagatcaactagtATGGAGGCGGCTGC	HR-PCR for pAN580
gfp-*Pik1-H4* R	tcctcgcccttgctcaccatggatccGCTAGTAGTTTCTGTTTGAATTTCAATATC	HR-PCR for pAN580
gfp-*Pik2-H4* F	ggagaggacagcccagatcaactagtATGGAGTTGGTGGTAGGTG	HR-PCR for pAN580
gfp-*Pik2-H4* R	tcctcgcccttgctcaccatggatccTGCAGTGACGATGCCATC	HR-PCR for pAN580
his-*Pik1-H4* F	GTGGACAGCAAATGGGTCGCGGATCCATGGAGGCGGCTGC	HR-PCR for pET28a
his-*Pik1-H4* R	CAGTGGTGGTGGTGGTGGTGCTCGAGGCTAGTAGTTTCTGTTTGAATTTCAAT	HR-PCR for pET28a
his-*Pik2-H4* F	GTGGACAGCAAATGGGTCGCGGATCCATGGAGTTGGTGGTAGGTGC	HR-PCR for pET28a
his-*Pik2-H4* R	CAGTGGTGGTGGTGGTGGTGCTCGAGTGCAGTGACGATGCCATCA	HR-PCR for pET28a
ad-*OsRRM2* F	GTACCAGATTACGCTCATATGATGGCGGCCACGCTCTT	HR-PCR for pGADT7
ad-*OsRRM2* R	CAGCTCGAGCTCGATGGATCCTCAATATTGTCTCCTAGGTG	HR-PCR for pGADT7
ad-*Pik1-H4*-CC F	acgacgtaccagattacgctcatatgATGGAGGCGGCTGCCATG	HR-PCR for pGADT7
ad-*Pik1-H4*-CC R	tatcgatgcccacccgggtggaattcTGTTTTTCTGAGCGGGAAATGCGG	HR-PCR for pGADT7
ad-*Pik1-H4*-HMA F	acgacgtaccagattacgctcatatgGGGCTAAAGCAAAAAATCGTGATCAAGG	HR-PCR for pGADT7
ad-*Pik1-H4*-HMA R	tatcgatgcccacccgggtggaattcCACATCTTTTTTTGCTTGGCTGACCT	HR-PCR for pGADT7
ad-*Pik2-H4*-NBS F	taccagattacgctcatatgAAGACGGTCATGGAGGAGCTTGAGGT	HR-PCR for pGADT7
ad-*Pik2-H4*-NBS R	tgcccacccgggtggaattcCAGGCCTTTTGTTGAACTTCCACGAT	HR-PCR for pGADT7
ad-*Pik2-H4*-LRR F	taccagattacgctcatatgAACTTGGCTCAAGTGAGATCACTGAC	HR-PCR for pGADT7

Continued Supplementary Table S1:

ad-*Pik*$_2$-*H4*-LRR R	tgcccacccgggtggaattcTCATGCAGTGACGATGCCATCAACAA	HR-PCR for pGADT7
ad-*OsRRM2*-1 F	taccagattacgctcatatgATGGCGGCCACGC	HR-PCR for pGADT7
ad-*OsRRM2*-1 R	agctcgagctcgatggatcctcaCCTCAGGTCGTCGGAG	HR-PCR for pGADT7
ad-*OsRRM2*-2 F	taccagattacgctcatatgGTCTTCGTCGGCAACCT	HR-PCR for pGADT7
ad-*OsRRM2*-2 R	agctcgagctcgatggatcctcaCCTGTTGGCCTCACCC	HR-PCR for pGADT7
ad-*OsRRM2*-3 F	taccagattacgctcatatgGTCTATGTAGGTAACCTTTCTTGGG	HR-PCR for pGADT7
ad-*OsRRM2*-3 R	agctcgagctcgatggatccTCAATATTGTCTCCTAGGTGGTT	HR-PCR for pGADT7
bd-*OsRRM2* F	TCAGAGGAGGACCTGCATATGATGGCGGCCACGCTCTT	HR-PCR for pGBKT7
bd-*OsRRM2* R	CCGCTGCAGGTCGACGGATCCTCAATATTGTCTCCTAGGTG	HR-PCR for pGBKT7
bd-*OsBIHD1* F	TCAGAGGAGGACCTGCATATGATGGCTACTTACTACTCGAG	HR-PCR for pGBKT7
bd-*OsBIHD1* R	CCGCTGCAGGTCGACGGATCCTTAGGCCACAAAATCATGCAT	HR-PCR for pGBKT7
bd-*Pik*$_2$-*H4* F	TCAGAGGAGGACCTGCATATGATGGAGTTGGTGGTAGGTGCTTCCGAAG	HR-PCR for pGBKT7
bd-*Pik*$_2$-*H4* R	CCGCTGCAGGTCGACGGATCCTCATGCAGTGACGATGCCATCAACAAAT	HR-PCR for pGBKT7
bd-*Pik*$_1$-*H4*-CC F	tgatctcagaggaggacctgcatatgATGGAGGCGGCTGCCATG	HR-PCR for pGBKT7
bd-*Pik*$_1$-*H4*-CC R	gcaggtcgacggatccccgggaattcTGTTTTTCTGAGCGGGAAATGCGG	HR-PCR for pGBKT7
bd-*Pik*$_1$-*H4*-HMA F	tgatctcagaggaggacctgcatatgCACATCTTTTTTTGCTTGGCTGACCT	HR-PCR for pGBKT7
bd-*Pik*$_1$-*H4*-HMA R	gcaggtcgacggatccccgggaattcCACATCTTTTTTTGCTTGGCTGACCT	HR-PCR for pGBKT7
bd-*Pik*$_2$-*H4*-LRR F	cagaggaggacctgcatatgAACTTGGCTCAAGTGAGATCACTGAC	HR-PCR for pGBKT7
bd-*Pik*$_2$-*H4*-LRR R	cgacggatccccgggaattcTCATGCAGTGACGATGCCATCAACAA	HR-PCR for pGBKT7
bd-*OsRRM2*-1 F	cagaggaggacctgcatatgATGGCGGCCACGC	HR-PCR for pGBKT7
bd-*OsRRM2*-1 R	cgctgcaggtcgacggatcctcaCCTCAGGTCGTCGGAG	HR-PCR for pGBKT7

Continued Supplementary Table S1:

bd-*OsRRM2*-2 F	cagaggaggacctgcatatgGTCTTCGTCGGCAACCT	HR-PCR for pGBKT7
bd-*OsRRM2*-2 R	cgctgcaggtcgacggatcctcaCCTGTTGGCCTCACCC	HR-PCR for pGBKT7
bd-*OsRRM2*-3 F	cagaggaggacctgcatatgGTCTATGTAGGTAACCTTTCTTGGG	HR-PCR for pGBKT7
bd-*OsRRM2*-3 R	cgctgcaggtcgacggatccTCAATATTGTCTCCTAGGTGGTT	HR-PCR for pGBKT7
gst-*OsRRM2* F	CGCGTGGATCCCCGGAATTCATGGCGGCCACGC	HR-PCR for pGEX-4T-1
gst-*OsRRM2* R	TCACGATGCGGCCGCTCGAGATATTGTCTCCTAGGTGGTTTCG	HR-PCR for pGEX-4T-1
oe-*OsRRM2* F	GTTTGGTGTTACTTCTGCAGATGGCGGCCACGC	HR-PCR for pOX-GFP
oe-*OsRRM2* R	CCCTTGCTCACCATACGCGTATATTGTCTCCTAGGTGGTTTCG	HR-PCR for pOX-GFP
cy-*Pik*$_1$-*H4* F	ttggagagaacacgggggactctagaATGGAGGCGGCTGC	HR-PCR for pUC-CE1R2R-nsI
cy-*Pik*$_1$-*H4* R	catcccgggagcggtaccctcgagGCTAGTAGTTTCTGTTTGAATTTCAAT	HR-PCR for pUC-CE1R2R-nsI
cy-*Pik*$_2$-*H4* F	ttggagagaacacgggggactctagaATGGAGTTGGTGGTAGGTG	HR-PCR for pUC-CE1R2R-nsI
cy-*Pik*$_2$-*H4* R	catcccgggagcggtaccctcgagTGCAGTGACGATGCCA	HR-PCR for pUC-CE1R2R-nsI
cy-*OsRRM2* F	ttggagagaacacgggggactctagaATGGCGGCCACGC	HR-PCR for pUC-CE1R2R-nsI
cy-*OsRRM2* R	catcccgggagcggtaccctcgagATATTGTCTCCTAGGTGGTTTCG	HR-PCR for pUC-CE1R2R-nsI
cfp-*Pik*$_1$-*H4* F	CTTCGAATTCTGCAGTCGACATGGAGGCGGCTGC	HR-PCR for pUC-CFP
cfp-*Pik*$_1$-*H4* R	ACTCTAGATCAGGTGGATCCCTAGCTAGTAGTTTCTGTTTGAATTTCAATAT	HR-PCR for pUC-CFP
t-Ubi F	TCTTTTTAGTGTGCATGTGTTCTCC	PCR test
t-*OsRRM2* R	GCAACTAGACACAATCATTCATCACC	PCR test
q-*PR1a* F	CGGAGAAGCAGTGGTACGA	qRT-PCR

Continued Supplementary Table S1:

q-PR1a R	TGATGAAGACGCCGAGGTC	qRT-PCR
q-PR1b F	GAACAACCTTGGCGAGAA	qRT-PCR
q-PR1b R	TTGGAGGCGTAGTCGTAG	qRT-PCR
q-PR10 F	CTTCATCGACGCCATTGA	qRT-PCR
q-PR10 R	GAACCTCTGACTTGATAATGTG	qRT-PCR
q-OsRRM2 F	GAAGGGGAGGTCTTGGAAGC	qRT-PCR
q-OsRRM2 R	ATCCATGTCAGCGCCATCAA	qRT-PCR
q-RAC1 F	ATGTGCTCTTCTGGATGGCT	qRT-PCR
q-RAC1 R	GAAAAGGTTGAAGCATGTGGCT	qRT-PCR
q-GAP1 F	TGCTATTGGTCTCAGCATTGGA	qRT-PCR
q-GAP1 R	TGTGTTGACCCGGGTTTGAT	qRT-PCR
q-CPK12 F	TCGAATTCACAGGACGGGAGA	qRT-PCR
q-CPK12 R	TTTTGCGTTTCATCTGCTTGC	qRT-PCR
q-EIL1A F	GCGACGATACTCCATTCGGT	qRT-PCR
q-EIL1A R	AGTACCAATTCGAGCCGTCA	qRT-PCR
q-APX8 F	AACCACTGCTCAAGCGTGTA	qRT-PCR
q-APX8 R	TCGTGCGAAATGGGTCTTCA	qRT-PCR
q-DJA6 F	AGTGGTGGATGAGCATTCGT	qRT-PCR
q-DJA6 R	ACAGAGACGCATCGAAGACC	qRT-PCR
q-RBOHB F	GACCCATTTCGCACGACCTA	qRT-PCR
q-RBOHB R	TGCAATGGATCATGGACATTCCT	qRT-PCR
q-Actin F	GATTGCCAAGGCTGAGTACG	qRT-PCR
q-Actin R	AAAAGAAGAAACAAGCAGGAT	qRT-PCR
q-OsPBZ1 F	CGCCGCAAGTCATGTCCTAA	qRT-PCR
q-OsPBZ1 R	CCACGATGTCCTTCTCCTTCTC	qRT-PCR
SELEX F	cgTAATACGACTCACTATAGGGATTACGCCAAGCTT	SELEX PCR
SELEX R	GGTACCCGGGGATCC	SELEX PCR
SELEX-Oligo	ggGATTACGCCAAGCTT$(N)_{40}$GGATCCCCGGGTACC	SELEX random primer

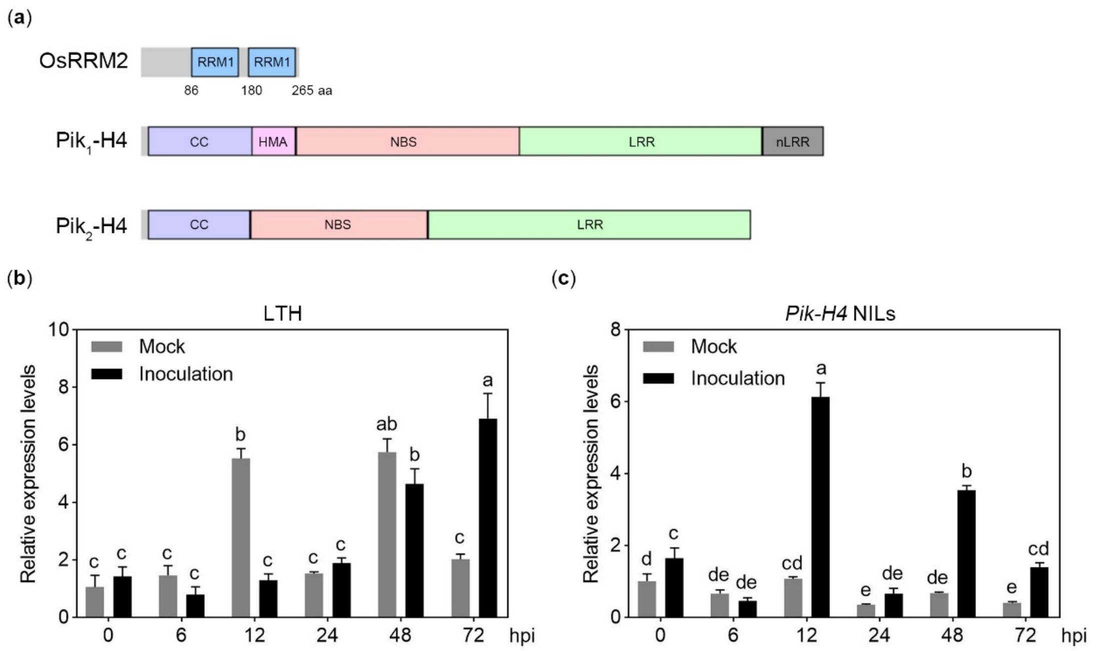

Supplementary Figure S1. OsRRM2 domain and expression pattern post-inoculation. (**a**) The protein plots of OsRRM2, Pik1-H4 and Pik2-H4. The OsRRM2 structure was analyzed by Pfam; (**b**) The expression pattern of *OsRRM2* upon rice blast inoculation in LTH; (**c**) The expression pattern of *OsRRM2* upon rice blast inoculation in *Pik-H4* NILs. The values are shown as mean ± SD (n=3). *β-actin* was tested as an internal reference, the 0 hpi mock treatment groups were regarded as the control groups. The lowercase letters (a, b, c, etc.) indicate the data were at *P*<0.01 level according to one-way ANOVA with Dunnett's test.

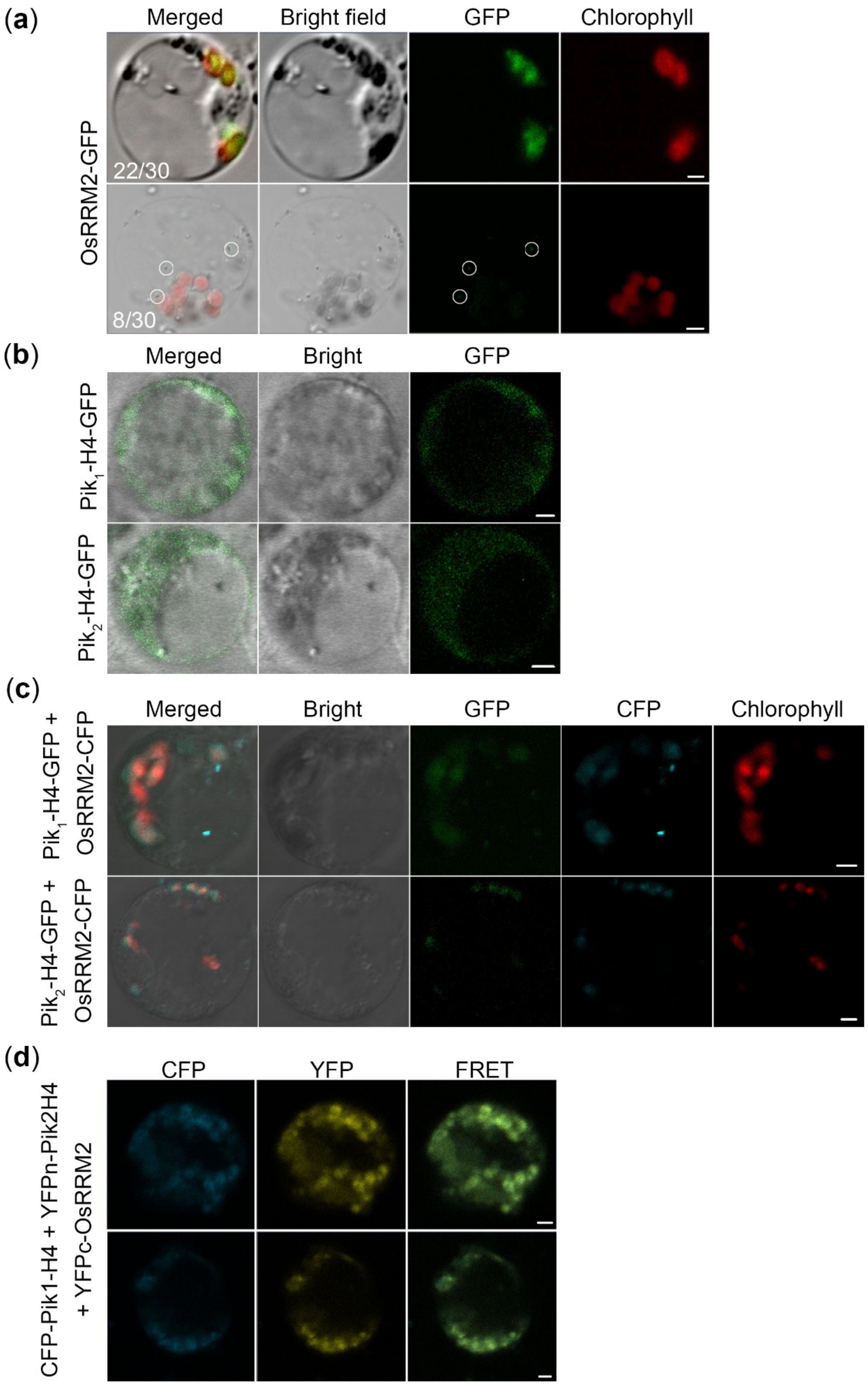

Supplementary Figure S2. Subcellular location of OsRRM2, Pik1-H4 and Pik2-H4. (**a**) Subcellular location of OsRRM2-GFP. The numbers represent the cells observed in this study.

n=30; (**b**) Subcellular location of Pik$_1$-H4-GFP and Pik$_2$-H4-GFP; (**c**) Co-location of OsRRM2-CFP with Pik$_1$-H4-GFP or Pik$_2$-H4-GFP; (**d**) FRET assays of CFP-Pik$_1$-H4, YFPn-Pik$_2$-H4 and YFPc-OsRRM2. The FRET channels were excited by 405 nm only. Scale bars = 5 μm.

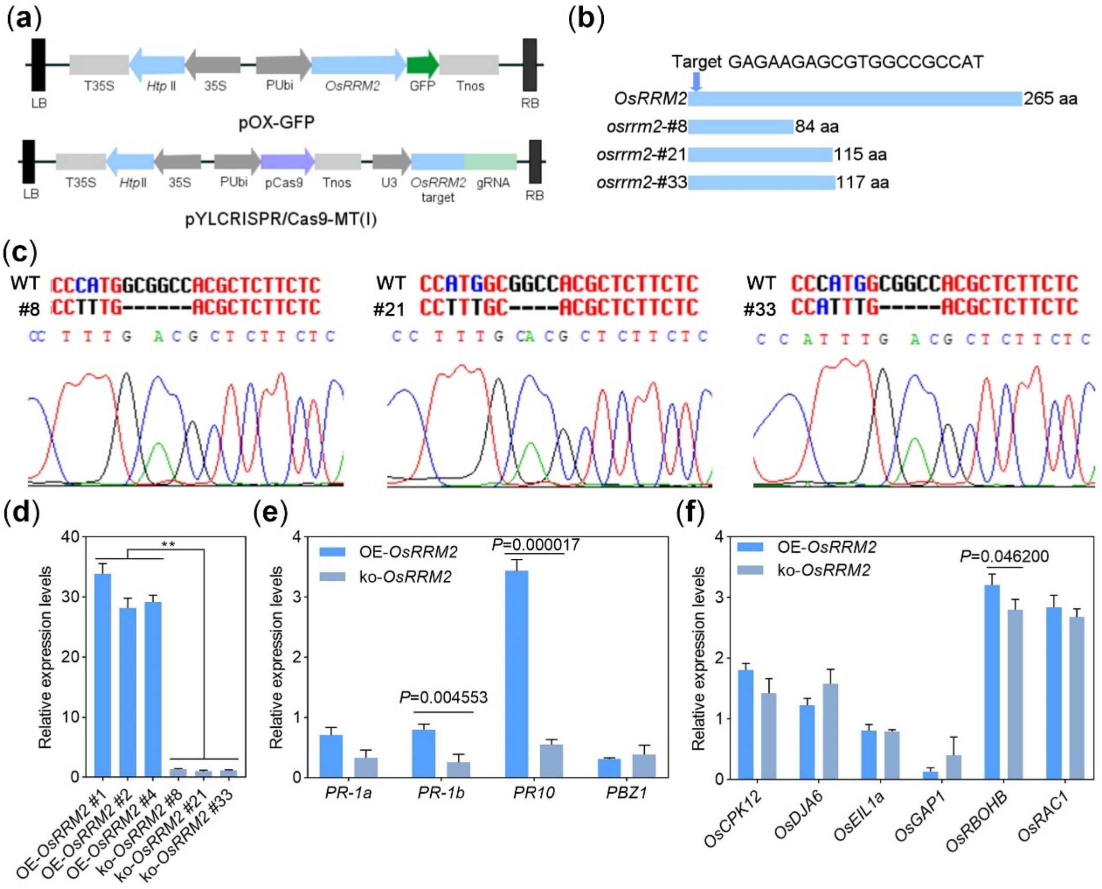

Supplementary Figure S3. Construction of the transgenic *OsRRM2* plants. (**a**) Plots of overexpression and knockout of *OsRRM2* plasmids; (**b**) The OsRRM2 CRISPR/Cas9 target and plots of *OsRRM2* mutants; (**c**) Alignments and chromatograms of *OsRRM2* mutants; (**d**) Relative expression levels of *OsRRM2* in transgenic plants. ** indicates the data were at *P*<0.01 level according to the Students' t-test; (**e**) The relative expression levels of resistance-related genes in *OsRRM2* transgenic plants; (**f**) The relative expression levels of ROS-related genes in *OsRRM2* transgenic plants. The values are shown as mean ± SD (n=3). *β-actin* was tested as an internal reference and the expression levels in *Pik-H4* NILs were regarded as the control groups.

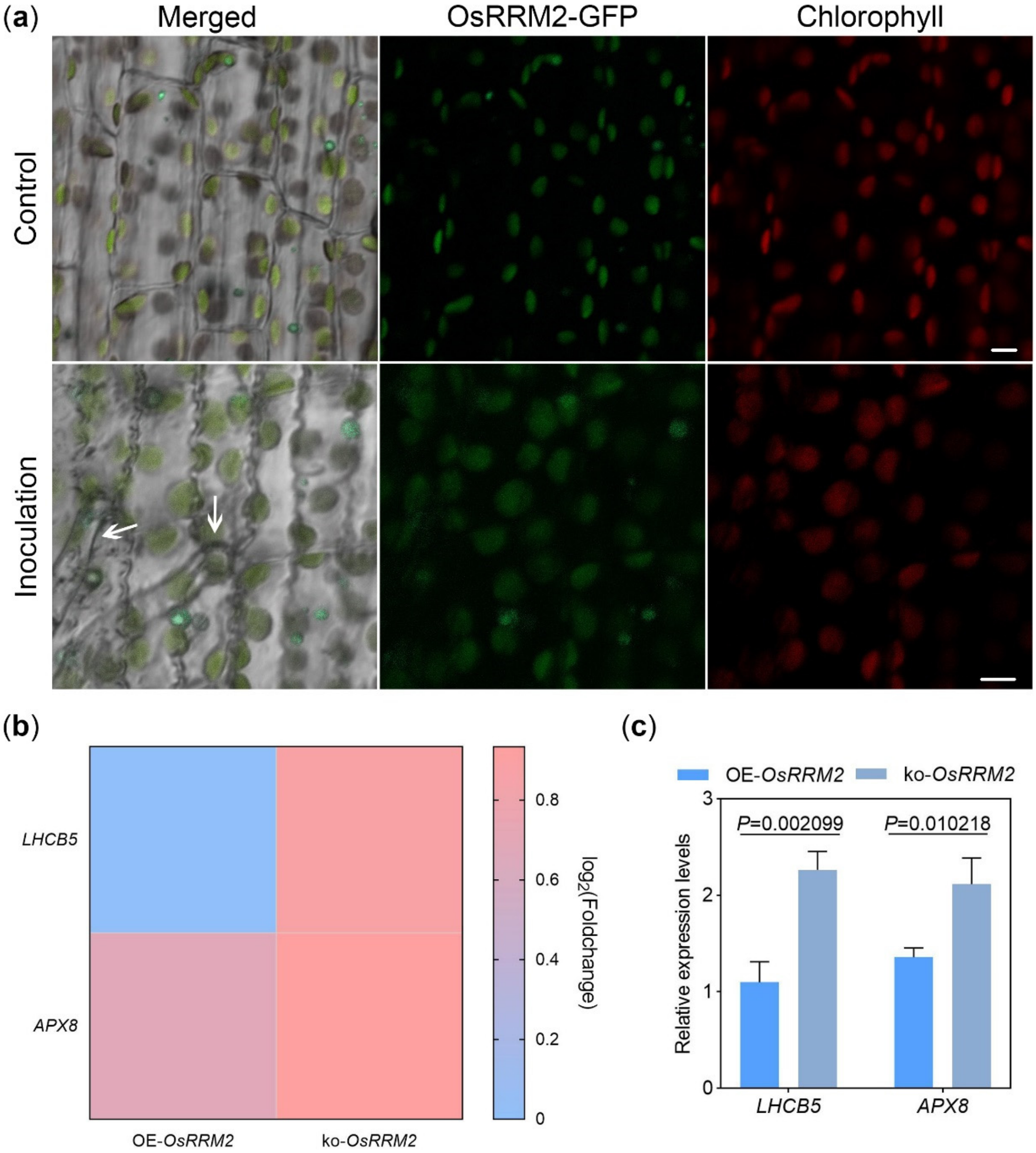

Supplementary Figure S4. OsRRM2 location upon rice blast infection and effect on chloroplast immune genes. (**a**) In vivo subcellular location of OsRRM2-GFP. The arrows indicate the hyphae of rice blast fungus. Scale bars = 5 μm; (**b**) Transcriptome analysis of chloroplast immune-related genes; (**c**) Relative expression levels of chloroplast immune-related genes. *β-actin* was tested as an internal reference and the expression levels in *Pik-H4* NILs were regarded as the control groups. The values are shown as mean ± SD (n=3). The data were compared according to the Students' t-test.

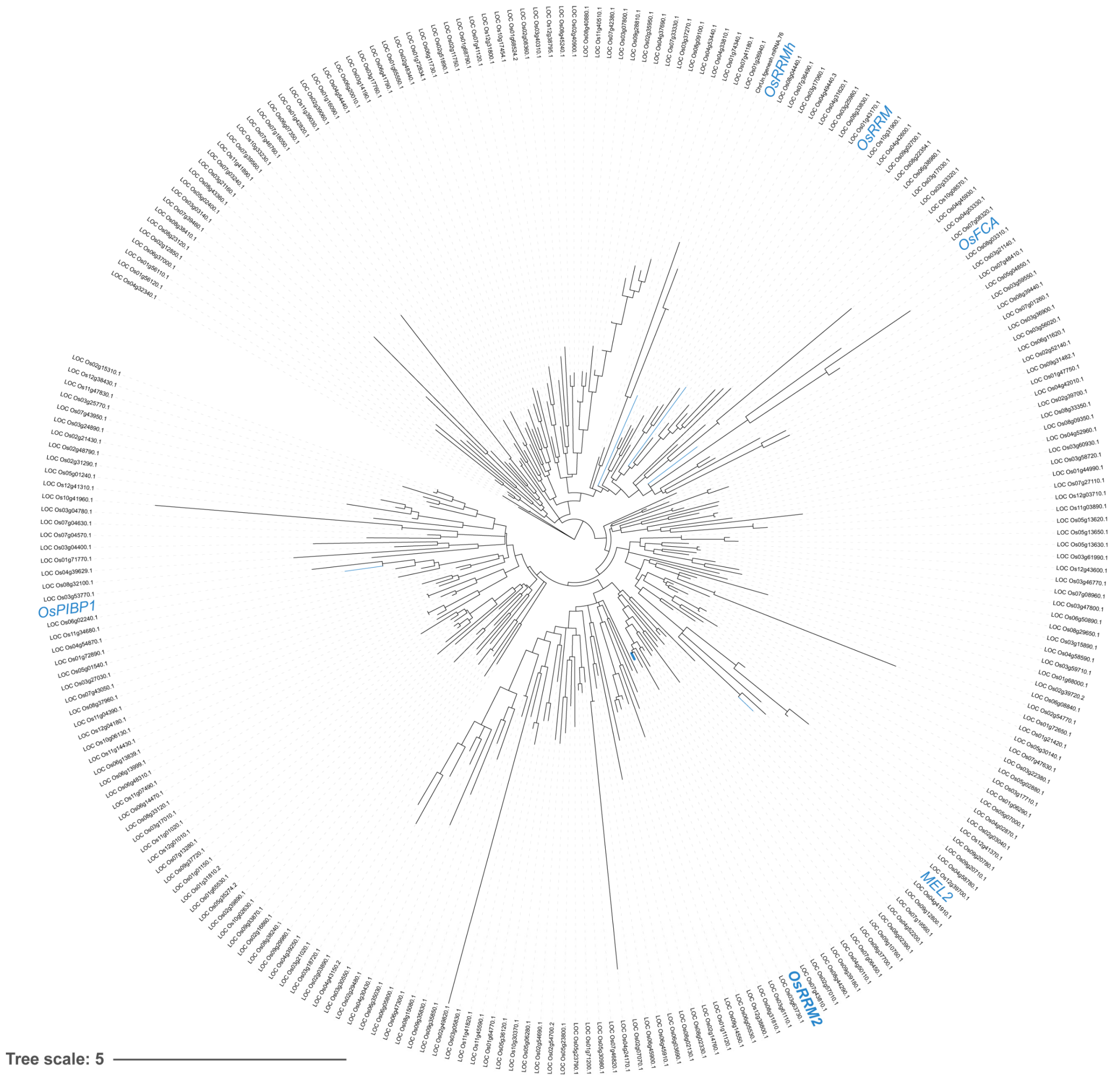

Supplementary Figure S5. Phylogenetic analysis of rice RRM family proteins. The reported RRM proteins are shaded in blue.
